# Orthodontic decompensation in skeletal Class III malocclusion: redefining the amount of movement assessed by Cone-Beam Computed Tomography

**DOI:** 10.1590/2177-6709.20.5.028-034.oar

**Published:** 2015

**Authors:** José Antonio Zuega Cappellozza, Fabio Pinto Guedes, Hugo Nary, Leopoldino Capelozza, Mauricio de Almeida Cardoso

**Affiliations:** 1 MSc in Orthodontics, Universidade Sagrado Coração (USC), Bauru, São Paulo, Brazil.; 2 PhD professor, Universidade Sagrado Coração (USC), Undergraduate and Graduate programs (specialization and MSc degrees) in Implantology, Bauru, São Paulo, Brazil. Head Professor, Branemark Institute, Bauru/SP.; PhD professor, Universidade Sagrado Coração (USC), Undergraduate and Graduate programs (specialization and MSc degrees), Bauru, São Paulo, Brazil.

**Keywords:** Tomography, Angle Class III malocclusion, Orthognathic surgery

## Abstract

**Introduction:**

Cone-Beam Computed Tomography (CBCT) is essential for tridimensional planning of orthognathic surgery, as it allows visualization and evaluation of bone structures and mineralized tissues. Tomographic slices allow evaluation of tooth inclination and individualization of movement performed during preoperative decompensation. The aim of this paper was to assess maxillary and mandibular incisors inclination pre and post orthodontic decompensation in skeletal Class III malocclusion.

**Methods:**

The study was conducted on six individuals with skeletal Class III malocclusion, surgically treated, who had Cone-Beam Computed Tomographic scans obtained before and after orthodontic decompensation. On multiplanar reconstruction view, tomographic slices (axial, coronal and sagittal) were obtained on the long axis of each incisor. The sagittal slice was used for measurement taking, whereas the references used to assess tooth inclination were the long axis of maxillary teeth in relation to the palatal plane and the long axis of mandibular teeth in relation to the mandibular plane.

**Results:**

There was significant variation in the inclination of incisors before and after orthodontic decompensation. This change was of greater magnitude in the mandibular arch, evidencing that natural compensation is more effective in this arch, thereby requiring more intensive decompensation.

**Conclusion:**

When routinely performed, the protocols of decompensation treatment in surgical individuals often result in intensive movements, which should be reevaluated, since the extent of movement predisposes to reduction in bone attachment levels and root length.

## Abstract (pt)

**Introdução:**

a tomografia computadorizada de feixe cônico (TCFC) é essencial para o planejamento tridimensional de cirurgias ortognáticas, pois permite a visualização e avaliação de estruturas ósseas e tecidos mineralizados. Os cortes tomográficos permitem avaliar a inclinação dos dentes e individualizar o movimento realizado durante a descompensação dentária.

**Objetivo:**

o objetivo do presente estudo foi avaliar a inclinação dos incisivos superiores e inferiores antes e depois da descompensação ortodôntica da má oclusão de Classe III esquelética.

**Métodos:**

o estudo foi realizado em seis indivíduos com má oclusão de Classe III esquelética que passaram por tratamento cirúrgico e apresentaram exames de TCFC feitos antes e depois da descompensação ortodôntica. A reconstrução multiplanar foi feita a partir dos cortes tomográficos (axial, coronal e sagital) adquiridos no eixo longitudinal de cada incisivo. O corte sagital foi usado para a realização de medições, e as referências usadas para avaliar a inclinação dentária foram o eixo longitudinal dos dentes superiores em relação ao plano palatino e o eixo longitudinal dos dentes inferiores em relação ao plano mandibular.

**Resultados:**

houve uma variação significativa entre a inclinação dos incisivos antes e depois da descompensação ortodôntica. A alteração foi maior na arcada inferior, evidenciando que a compensação natural é mais efetiva nessa arcada, o que exige uma descompensação mais extensa.

**Conclusão:**

quando realizados rotineiramente, os protocolos de tratamento para descompensação em pacientes cirúrgicos geralmente resultam em movimentação extensa, que deve ser reavaliada, já que a extensão do movimento predispõe à perda de inserção e à redução do comprimento da raiz.

## INTRODUCTION

Assessing images by means of Cone-Beam Computed Tomography (CBCT) has been a method widely used in Dentistry with little restriction when applied to adults. In Implantology, it is used to measure bone height and thickness;^1^ in maxillofacial surgery, for extractions, access for surgically-assisted tooth eruption, reduction in fractures and for removal of pathologies;^2^ in orthognathic surgery, it is employed to provide adequate tridimensional planning;^3^ and in Orthodontics, it is used to assess buccal and lingual bone plates,^4^^,^ 5 ^,^ 6 especially the adequate location of unerupted teeth, in addition to determining surgical traction and indication for extraction.^5^

The multiple possibilities of observation and evaluation of bone structures and mineralized tissues offered by analysis of tomographic slices allow this type of examination to be used to assess tooth inclination in Orthodontics,^6^^,^ 7 especially for surgical patients whose orthodontic treatment often requires movements of greater magnitude and risk. This type of examination allows assessment of each tooth with better image quality, and also allows analysis of occasional differences in their behavior. The images obtained by this new method demonstrate that the condition of teeth, especially maxillary and mandibular incisors located at the anterior region where there is greater compensation, is more fragile than what was previously believed.^8^^,^ 9 In other words, the relationship between dental root and alveolar bone is critical.

For this reason, CBCT is recommended to assess tooth inclination with a view to investigating the magnitude of preoperative orthodontic decompensation of maxillary and mandibular incisors. The aim of this paper was to assess maxillary and mandibular incisors inclination pre and post orthodontic decompensation in skeletal Class III malocclusion patients.

## MATERIAL AND METHODS

The study was approved by Universidade Sagrado Coração (USC) Institutional Review Board under protocol # 153/11.

### Sample selection

In the present study, a total of 15 individuals with skeletal Class III malocclusion and unpleasant face were selected. Patients had been submitted to full-face CBCT for planning of orthognathic surgery, and treated by orthodontic specialists at Instituto Brånemark in Bauru, São Paulo, Brazil. In selecting the sample, the following exclusion criteria were applied: 1) missing teeth in the maxillary and/or mandibular anterior region; 2) previous orthodontic treatment; 3) history of periodontal disease; 4) severe facial asymmetry.

The study sample included six adult individuals (four males and two females), with age ranging from 20 years and 6 months to 40 years and 1 month old, with mean age of 27 years and 6 months old.

The protocol of orthodontic decompensation was the same for all individuals in the sample and consisted of maxillary alignment and leveling avoiding protrusion, and mandibular alignment and leveling accepting protrusion.

Treatment was conducted with fixed orthodontic appliances with 0.022 x 0.030-in slots (Abzil-3M, Capelozza I prescription, São José do Rio Preto, Brazil) for both maxillary and mandibular arches. The appliances were initially bonded to the mandibular arch, which required greater movement, since these teeth undergo more significant decompensation. The sequence of archwires used was: 0.018-in NiTi; 0.016-in stainless steel; 0.018-in stainless steel; 0.020-in stainless steel; 0.019 x 0.025-in Titanal XR; 0.019 x 0.025-in stainless steel.

Only three patients were treated with extraction of maxillary first premolars, which aimed to correct severe crowding without causing protrusion.

The mean time of decompensation treatment was 25 months, ranging from 12 to 37 months, according to the complexity of the initial malocclusion.

### Cone-Beam Computed Tomography

All individuals included in the sample were submitted to Cone-Beam Computed Tomography (CBCT) before treatment and after placement of 0.019 x 0.025-in stainless steel archwires in the maxillary and mandibular arches (at least 30 days later). CBCT scans were obtained on i-CAT (Imaging Sciences International, Hatfield, USA), set at 120 KvP, 8 Ma, exposure time of 40 seconds, "extended face" protocol with 22 cm of FOV, and voxel of 0.4 mm; a protocol commonly used for surgical planning.

In order to standardize head positioning in the three planes of space, the individuals sat for the examination with the Frankfort plane parallel to the ground and the midsagittal plane perpendicular to the ground. ConeBeam Computed Tomography scans were obtained in DICOM format (Digital Imaging and Communication in Medicine), which allows their manipulation in software for observation of volumetric images.

### Selection of images for measurement

The measurement taking method used in the present study was initially proposed by Kim, Park and Kook^9^ and adapted for InVivoDental^5^ software (Anatomage, San Jose, CA, USA) in which maxillary and mandibular incisors inclination before and after orthodontic decompensation was analyzed. Measurements were analyzed on sagittal slices 1-mm in thickness, obtained as follows.

**Figure 1:**
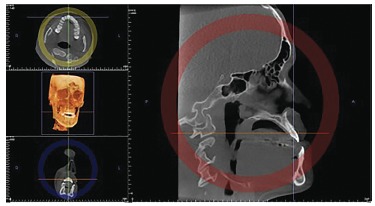
- Multiplanar reconstruction.

**Figure 2:**
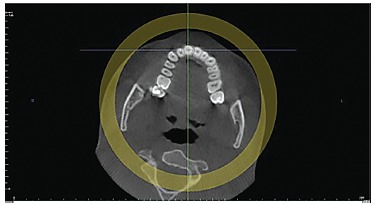
- Axial tomographic slice.

**Figure 3:**
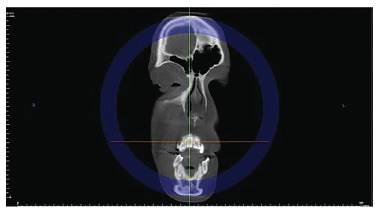
- Coronal slice.

**Figure 4:**
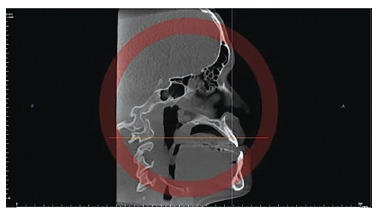
- Sagittal slice.

On multiplanar reconstruction view (Fig 1), the individual's head was manipulated, so as the vertical reference line was superimposed to the long axis of each tooth. To this end, the slice was analyzed in three directions: axial, coronal and sagittal (Figs 2, 3 and 4). At this stage, it is important to highlight the possibility of tridimensional observation for better visualization of the slice to be obtained.

The sagittal slice was used for measurement taking, whereas the long axis of maxillary teeth with the palatal plane and mandibular teeth with the mandibular plane were used as reference to assess tooth inclination.

In the maxillary arch, the slices were selected as follows: initially, maxillary right lateral incisor, followed by maxillary right and left central incisors, ending by the maxillary left lateral incisor. In the mandibular arch, the slices were initially selected by the mandibular left lateral incisor, followed by mandibular left and right central incisors, ending by the mandibular right lateral incisor.

**Figure 5:**
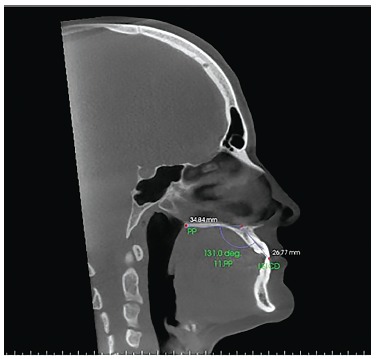
- Sagittal slice with the individual's head positioned so as the horizontal reference line was superimposed to the palatal plane for measurement of maxillary right central incisor inclination before orthodontic decompensation.

**Figure 6:**
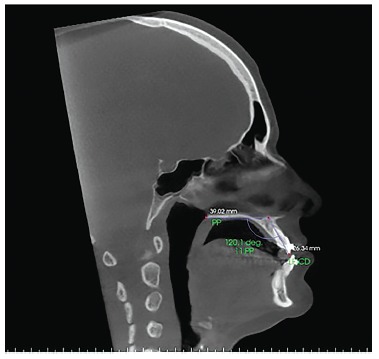
- Sagittal slice with the individual's head positioned so as the horizontal reference line was superimposed to the palatal plane, for measurement of maxillary right central incisor inclination after orthodontic decompensation.

**Figure 7:**
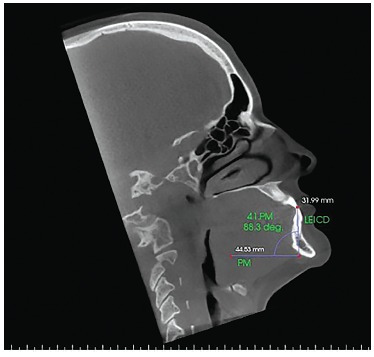
- Sagittal slice with the individual's head positioned so as the horizontal reference line was superimposed to the mandibular plane for measurement of mandibular right central incisor inclination before orthodontic decompensation.

**Figure 8:**
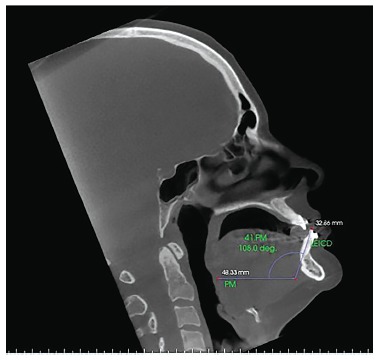
- Sagittal slice with the individual's head positioned so as the horizontal reference line was superimposed to the mandibular plane for measurement of mandibular right central incisor inclination after orthodontic decompensation.

The individual's head was rotated, so as the horizontal reference line was superimposed to the palatal plane to assess maxillary teeth inclination (Figs 5 and 6) and superimposed to the mandibular plane to assess mandibular teeth inclination (Figs 7 and 8).

## RESULTS

Assessment by means of Cone-Beam Computed Tomography (CBCT) of six Class III malocclusion individuals submitted to orthodontic decompensation for orthognathic surgery revealed significant changes in tooth inclination.

Despite variations in magnitude, changes were observed in the position of incisors before and after orthodontic decompensation, with palatal movement of maxillary incisors and buccal movement of mandibular incisors, as presented in Tables 1 and 2.

**Table 1:** - Measurement of maxillary incisors inclination pre- and post-orthodontic decompensation.

**Patient**		**Pre-decompensation**		**Maxillary incisors inclination** ** Post-decompensation**		**Results**						
	**12**	**11**	**21**	**22**	**12**	**11**	**21**	**22**	**12**	**11**	**21**	**22**
1	114.3^o^	114.8^o^	115.3^o^	113.3^o^	113.0^o^	107.9^o^	113.4^o^	114.1^o^	- 1.4^o^	- 6.9^o^	- 1.9^o^	0.8^o^
2	128.9^0^	131.0^0^	129.0^0^	126.5^0^	11.8^0^	120.1^0^	118.1^0^	109.7^0^	-10.9^0^	- 9.9^0^	- 10.9	-16.8^0^
3	122.4^0^	119.0^0^	118.1^0^	116.6^0^	123.1^0^	110.0^0^	116.1^0^	120.5^0^	0.7^0^	- 9.0^0^	- 2.0^0^	3.9^0^
4	125.7^0^	124.3^0^	123.5^0^	127.0^0^	125.5^0^	122.3^0^	119.1^0^	122.0^0^	-0.2^0^	-2.0^0^	-4.4^0^	-5.0^0^
5	117.9^0^	112.9^0^	114.2^0^	106.9^0^	115.5^0^	114.7^0^	112.5^0^	117.3^0^	-2.4^0^	1.8^0^	-1.7^0^	10.4^0^
6	122.7^0^	125.1^0^	128.9^0^	114.4^0^	123.8^0^	128.0^0^	129.1^0^	119.4^0^	1.1^0^	2.9^0^	0.2^0^	5.0^0^

**Table 2:** - Measurement of mandibular incisors inclination pre- and post-orthodontic decompensation.

**Patient**		**Pre-decompensation**		**Mandibular incisors inclination** ** Post-decompensation**		**Results**						
	**32**	**31**	**41**	**42**	**32**	**31**	**41**	**42**	**32**	**31**	**41**	**42**
1	79.7^0^	74.2^0^	75.5^0^	77.0^0^	89.1^0^	89.7^0^	89.0^0^	91.3^0^	9.4^0^	15.5^0^	13.5^0^	14.3^0^
2	83.3^0^	87.6^0^	88.3^0^	89.1^0^	100.0^0^	104.5^0^	108.0^0^	105.9^0^	16.7^0^	16.9^0^	19.7^0^	16.8^0^
3	78.3^0^	83.2^0^	79.5^0^	78.9^0^	85.7^0^	86.8^0^	88.3^0^	88.4^0^	7.4^0^	3.6^0^	8.8^0^	9.5^0^
4	67.6^0^	67.1^0^	67.1^0^	64.9^0^	74.6^0^	77.2^0^	84.2^0^	80.9^0^	7.0^0^	10.1^0^	17.1^0^	16.0^0^
5	72.8^0^	70.1^0^	69.8^0^	66.4^0^	87.6^0^	87.3^0^	89.1^0^	87.5^0^	14.8^0^	17.2^0^	19.3^0^	21.1^0^
6	80.2^0^	76.0^0^	74.3^0^	83.7^0^	96.0^0^	94.9^0^	96.7^0^	94.7^0^	14.8^0^	18.9^0^	22.4^0^	11.0^0^

## DISCUSSION

Orthodontic-surgical treatment is recommended for individuals presenting dentoskeletal deformities of sufficient magnitude so as to render their faces unpleasant.^10^^,^ 11

Natural compensation present in the dental arches occurs as an attempt to allow function. It is frequently observed in individuals with skeletal Class III malocclusion, probably because they go through a stage of normality and compensate slowly throughout the process of growth.12,13,14,15

Orthodontic decompensation in skeletal Class III malocclusion aims to achieve lingual inclination of maxillary incisors, occasionally requiring extraction of maxillary premolars, while mandibular incisors should be buccally inclined.^10^^,^ 11

The treatment protocols employed for decompensation of the individuals included in the present sample were standardized according to the classic protocol and under approval of the maxillofacial surgeon.

The advent of CBCT allowed better visualization of the area of interest and teeth involved in the process, especially incisors. Also, each tooth may be investigated individually, different from the conventional examination used in Orthodontics for that purpose. It is known that lateral cephalograms do not identify the incisor of which analysis was based on an unspecific and combined image of all teeth in the anterior region.^8^ Several studies have been conducted to analyze the quality of images obtained by CBCT examinations. Menezes et al^16^ assessed different examinations with voxel sizes of 0.2, 0.3, and 0.4 mm, and concluded that the morphology of dental and bone structures was relatively accurate on CBCT scans. These findings allowed CBCT scans with a voxel size of 0.4 mm, as used in the present study, to be used to measure the aforementioned proposed structures. The protocol adopted for the tomographic scans was the most frequently used for tridimensional planning of orthognathic surgery, with FOV of 22 cm and voxel size of 0.4 mm.^17^

The orthodontic decompensation analyzed triggered changes in incisors inclination, especially mandibular incisors, as it is usually observed and considering that compensation is more intensive in this arch.^11^ In the maxillary arch, central incisors presented a mean reduction in inclination of 2.5°, compared to 1.2° for lateral incisors. In the mandibular arch, a mean increase of 15.2° was observed for central incisors and 13.6° for lateral incisors; a significant movement and the main factor accounting for increased negative overjet. This intention transformed into action is probably related to the time when the expression of decompensation had significant correlation with the quantity and quality of surgery, especially in sagittal discrepancies, such as skeletal Class III malocclusions. It is noteworthy that sample size limited the use of statistical analysis.

The evolution of procedures and techniques allows better individualization of therapeutic goals for decompensation. Therefore, currently, evidence of the relationship between teeth and supporting bone, as found in the individuals taking part in the present study, are weak and the finding of correlation between movement at the area (decompensation) and attachment loss and root shortening^9^ assures that reassessment of these procedures seems necessary and possible. In the present sample, significant bone attachment loss and root shortening were observed,^18^which disagrees with the belief of extensive quantity of movement and treatment time required for its occurrence. That is to say, the extent of decompensation, especially in the mandibular arch, should not rely on the quantity of crowding and lingual inclination, both of which are caused by compensation during growth.

Whenever possible, and in individuals followed-up during growth, procedures aimed to minimize compensation, such as interruption of natural compensatory movement (limiting agent such as lingual retainers) and/or extractions, should be recommended.^13^ In individuals in which compensation has already been established, and who have been assessed during adulthood, individualized therapeutic goals should be determined.^11^^,^ 13 The amount of crowding and compensatory inclinations should be considered, in addition to the risk inherent to movements to be adopted for their elimination, with greater emphasis on the magnitude, treatment time and gingival phenotype,^18^ and special attention to the mandibular arch.

It should be remembered that the surgeon may allow correction of skeletal relationships, in which there may be reasons to minimize decompensation. Under the surgeon's approval, more consistent decompensation, aiming to maintain bone attachment and root length, may be planned while respecting the scientific evidence provided by computed tomographic images.

## CONCLUSIONS

Treatment protocols for orthodontic decompensation in individuals with skeletal Class III deformities determined movement of anterior teeth. CBCT revealed, in a more consistent and individualized manner, significant changes in the inclination of mandibular incisors, which may raise concerns and have some impact in the long-term. This evaluation clearly demonstrated that treatment protocols should be individualized, aiming to achieve movements of smaller magnitude whenever possible, especially for mandibular incisors.
